# Phototropin monitors actual temperature, not temperature difference, to regulate temperature-dependent chloroplast movement via *cis*–*trans* autophosphorylation mode switching in *Marchantia polymorpha*

**DOI:** 10.1007/s00425-026-04923-1

**Published:** 2026-01-17

**Authors:** Minoru Noguchi, Tatsushi Fukushima, Saki Wakasugi, Yutaka Kodama

**Affiliations:** 1 Center for Bioscience Research and Education, Utsunomiya University, Tochigi, 321-8505 Japan; 2 Graduate School of Regional Development and Creativity, Utsunomiya University, Tochigi, 321-8505 Japan

**Keywords:** Actual temperature, Chloroplast movement, Cold-avoidance response, *Marchantia polymorpha*, Phototropin, Thermosensing

## Abstract

**Main conclusion:**

In liverworts, phototropin senses the actual temperature rather than temperature differences and switches from *cis*- to *trans*-autophosphorylation to trigger the cold-avoidance response of chloroplast movement.

**Abstract:**

Blue-light (BL)-induced chloroplast movement in plant cells is temperature-dependent. At standard growth temperatures, chloroplasts move toward weak BL-irradiated regions (accumulation response), maximizing photoreception, whereas at lower temperatures they move away from the irradiated area (cold-avoidance response), reducing photodamage. This temperature-dependent switch in the chloroplast response is mediated by phototropin (phot), a BL receptor and thermosensor, which contains a kinase domain and undergoes *cis*- and *trans*-autophosphorylation in response to BL and temperature. Under weak BL conditions, phot autophosphorylates in *cis* at standard growth temperatures and in both *cis* and *trans* at lower temperatures. However, it remains unclear whether phot senses actual temperatures or relative temperature changes to regulate chloroplast movement via autophosphorylation. In this study, we analyzed phot-mediated chloroplast movement in the liverwort *Marchantia polymorpha* under varying temperature conditions. We determined that chloroplast movement responds to actual temperatures rather than temperature differences and confirmed that phot is responsible for sensing actual temperatures *in planta*. Phot continuously monitors the actual temperature and increases its autophosphorylation levels as temperature decreases. The threshold temperature for the transition between the accumulation response and the cold-avoidance response corresponds to that for the switch from *cis*- to *trans*-autophosphorylation of phot. Our findings reveal that phot serves as an actual temperature sensor *in planta* to regulate chloroplast movement through autophosphorylation mode switching.

**Supplementary Information:**

The online version contains supplementary material available at 10.1007/s00425-026-04923-1.

## Introduction

To acclimate to changes in the ambient environment, plant cells sense those changes and generate intracellular signals that induce the appropriate physiological responses. One such response is chloroplast movement: the changes in the subcellular positions of chloroplasts that occur in response to changes in light and temperature (Senn 1908). The accumulation response, in which chloroplasts move toward the light-irradiated region of the cell, is induced under weak blue-light (wBL) conditions at standard growth temperatures (e.g., 22 °C) (Sakai et al. 2001; Wada et al. 2003). Conversely, the cold-avoidance response, in which chloroplasts move away from the light-irradiated region of the cell, is induced under wBL conditions at lower temperatures (e.g., 5 °C) (Kodama et al. 2008; Fujii et al. 2017). The accumulation and cold-avoidance responses enhance photosynthesis and suppress photoinhibition, respectively (Fujii et al. 2017; Gotoh et al. 2018), optimizing photosynthetic efficiency under fluctuating temperatures.

Chloroplast movement is mediated by the blue light (BL) receptor kinase phototropin, which contains flavin mononucleotide (FMN) as a chromophore (Christie 2007). The phototropin gene, apoprotein, and holoprotein are termed *PHOT*, PHOT, and phot, respectively (Briggs et al. 2001). Phot comprises two light-oxygen-voltage (LOV1 and LOV2) domains bound to FMN at its N-terminus and a serine/threonine kinase domain at its C-terminus (Christie 2007). Both LOV1 and LOV 2 sense BL and regulate the activity of the kinase domain, with the LOV2 domain playing a central role in this process (Matsuoka and Tokutomi 2005; Kato et al. 2021). In the dark, phot is inactive, with the LOV domains noncovalently bound to FMN (Christie 2007). Upon BL irradiation, the LOV domains form a covalent bond with FMN, which defines the photoactive state that activates the kinase domain (Christie 2007). The kinase activity of phot is required to induce chloroplast movement (Inoue et al. 2008, 2011). The photoactivated LOV domains revert to the inactive state through a thermal reversion reaction. This thermal reversion process is the mechanism by which phot senses temperatures to regulate chloroplast movement in the liverwort *Marchantia polymorpha* and in *Arabidopsis thaliana* as well as to regulate phototropism and stomatal opening in Arabidopsis (Fujii et al. 2017; Noguchi et al. 2025a). In the case of chloroplast movement, for example, low temperatures suppress thermal reversion, increasing the amount of photoactivated LOV2 domain and enhancing kinase activity to induce the cold-avoidance response (Okajima et al. 2012; Fujii et al. 2017). In this way, phot functions as a BL-dependent thermosensor.

*M. polymorpha* has a single copy of *PHOT* (Mp*PHOT*), which mediates both the accumulation and cold-avoidance responses (Komatsu et al. 2014; Fujii et al. 2017). In *M. polymorpha*, the accumulation response is induced under wBL conditions (25 µmol m^−2^ s^−1^) at 22 °C, whereas the cold-avoidance response is triggered by decreasing the temperature from 22 °C to 5 °C under the same light conditions (Fujii et al. 2017). We recently determined that Mpphot employs two autophosphorylation modes: *ci*s-autophosphorylation, which occurs within a single phot protein; and *trans*-autophosphorylation, which requires intermolecular interactions between two phot proteins (Noguchi et al. 2025b). Under wBL conditions at 22 °C, Mpphot autophosphorylates in *cis*; at 5 °C, both *ci*s- and *trans*-autophosphorylation occur (Noguchi et al. 2025b). The low-temperature-induced *trans*-autophosphorylation increases the overall level of Mpphot autophosphorylation and is required to induce the cold-avoidance response (Noguchi et al. 2025b). However, it remains unclear whether these responses involve phot sensing the actual temperature of 5 °C or the relative temperature, i.e., the 17 °C difference between 22 °C and 5 °C.

In this study, we aim to determine whether phot senses actual temperature or relative temperature to switch between the accumulation and the cold-avoidance responses in *M. polymorpha*. We demonstrate that phot continuously monitors actual temperatures to induce the appropriate chloroplast movement through autophosphorylation mode switching.

## Materials and methods

### Plant materials and culture conditions

*M. polymorpha* thalli and gemmalings were asexually cultured on half-strength B5 medium with 1% (w/v) agar (BOP; SSK Sales, Osaka, Japan) under approximately 70 µmol m^−2^ s^−1^ of continuous white light (FL40SW; NEC Co., Tokyo, Japan) (Ogasawara et al. 2013). The male strain Tak-1 was used as the wild type (WT). Mpphot knockout *M. polymorpha* (Mp*phot*^*KO*^) expressing an Mpphot mutant carrying a valine-to-threonine substitution at position 594 (Mpphot^V594T^/Mp*phot*^*KO*^) was described previously (Fujii et al. 2017). Transformants expressing a Citrine (Cit)-fused kinase-inactive (KI) Mpphot mutant (Mpphot-KI-Cit) in the WT background (Mpphot-KI-Cit/WT #1) were also described previously (Noguchi et al. 2025b).

For BL irradiation, gemmalings were incubated in a temperature-regulated incubator (IJ100; Yamato Scientific Co., Ltd., Tokyo, Japan) supplied with light-emitting diode panels (ISL-150 × 150-RHB; CCS Inc., Kyoto, Japan) controlled by a constant current power supply (ISC-201-2; CCS Inc.). Light intensity was measured using a LI-250A light meter (LI-COR Inc., Lincoln, NE, USA).

### Observation of chloroplast movement

Chloroplast positions were analyzed in 1-day-old gemmalings. The gemmalings were cultured under 25 µmol m^−2^ s^−1^ (BL25) or 100 µmol m^−2^ s^−1^ (BL100) of BL at various temperatures. Chloroplast positions were observed by monitoring chlorophyll fluorescence using a stereo fluorescence microscope (MZ16F or M165 FC; Leica Microsystems) equipped with an excitation filter at 480/40 nm and a long-pass barrier filter at 510 nm. The images were captured using a DP73 camera system (Olympus Co.), and chloroplast positions were evaluated by the P/A ratio method (Fig. S1) (Kodama et al. 2008).

### Statistical method

All statistical analyses were performed using GraphPad Prism Version 8.4.3 (https://www.graphpad.com).

### Analysis of the photoactivated LOV2 domain

Maltose-binding protein (MBP)-tagged recombinant LOV2 domains from Mpphot (MBP-Mpphot-LOV2) were produced in *Escherichia coli* and purified as described previously (Fujii et al. 2017; Yong et al. 2024). The percentage of photoactivated LOV2 domains under BL conditions was calculated based on the fluorescence detected from inactive LOV2 domains (Kato et al. 2021). The measurements were conducted using a fluorescence spectrophotometer (F2700; Hitachi High-Tech Co., Tokyo, Japan). The sample temperature was regulated via incubation in a chiller unit (NCB-1200; EYELA, Tokyo, Japan) equipped with a water-cooled cell holder (catalog no. 210–2111; Hitachi High-Tech Co.) (Kato et al. 2021). After the recombinant proteins were incubated in the dark at 2 °C, 7 °C, 12 °C, 17 °C, or 22 °C for 30 min, fluorescence at 525 nm was measured every 2 s for 30 min upon excitation at 470 nm with the temperature maintained. The voltage of the photomultiplier was set to 400 V for excitation of the LOV2 domain. The maximum fluorescence measured immediately after excitation light irradiation was defined as the state in which all LOV2 domains were inactive (*F*_a_). The fluorescence gradually decreased with increasing LOV2 activation, and the mean fluorescence from 1,200 s to 1,800 s after irradiation with excitation light was defined as the state in which active LOV2 and inactive LOV2 reached equilibrium (*F*_e_). The percentage of photoactivated LOV2 domains was calculated as [(*F*_a_-* F*_e_)/* F*_a_] × 100 (Noguchi et al. 2025a).

### Protein extraction from *M. polymorpha*

Three-day-old gemmalings were incubated under BL25 conditions at 2 °C, 7 °C, 12 °C, 17 °C, or 22 °C for 1 day. As a control, 3-day-old gemmalings were incubated in the dark at 22 °C for 1 day (Dark 22 °C). The gemmalings were collected, snap frozen in liquid nitrogen, and ground in 1 × SDS sample buffer (50 mM Tris–HCl [pH 6.8], 2% SDS, 10% glycerol, 3% 2-mercaptoethanol) to extract total proteins. The samples were centrifuged at 14,000 g to remove debris, incubated at 95 °C for 3 min, and protein concentrations were measured using an XL-Bradford assay (APRO Science Group, Tokushima, Japan).

### Analysis of Mpphot autophosphorylation levels

To analyze Mpphot autophosphorylation levels, a gel electrophoresis mobility shift assay was performed as previously described (Noguchi et al. 2025b). In brief, proteins were separated by SDS-PAGE using an 8% (w/v) polyacrylamide gel (acrylamide:N,N'-methylenebisacrylamide = 29.9:0.1, v/v) and transferred to a polyvinylidene difluoride membrane (Immobilon-P; Merck). Mpphot protein was detected using anti-MpPHOT antibodies (Hirano et al. 2024) as primary antibodies and goat anti-rabbit IgG (H + L) as secondary antibodies (catalog no. 32460; Thermo Fisher Scientific Inc.), both at a dilution of 1:2000. Chemiluminescent signals were detected using ECL-Select reagent (Cytiva, Tokyo, Japan) and a LuminoGraph III chemiluminescence imaging system (ATTO Co., Tokyo, Japan). Band intensity histograms were created using ImageJ software (https://imagej.net/ij/), and the mobility shift level was calculated based on the histograms. To quantify the mobility shift of endogenous Mpphot in WT cells, the band positions of dark at 22 °C and BL25 at 22 °C samples were defined as 0 and 1, respectively, and the relative band positions of the other samples (BL25 at 2 °C, 7 °C, 12 °C, and 17 °C) were calculated. To quantify the *trans*-autophosphorylation of Mpphot-KI-Cit in Mpphot-KI-Cit/WT cells, the band positions of dark at 22 °C and BL25 at 2 °C samples were defined as 0 and 1, respectively, and the relative band positions of the other samples (BL25 at 7 °C, 12 °C, 17 °C, and 22 °C) were calculated.

## Results

### Temperature-dependent transition between the accumulation and cold-avoidance responses

**Fig. 1:**
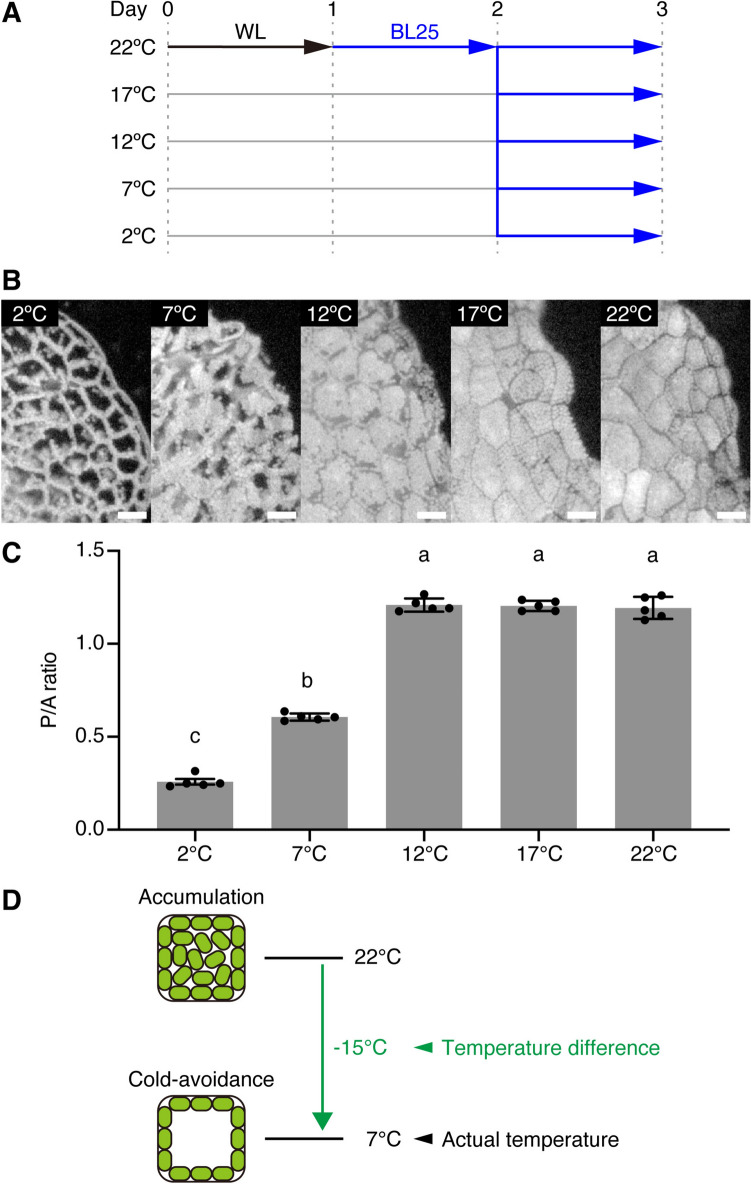
Chloroplast positioning in *M. polymorpha* at various temperatures under BL25 conditions. **A** Diagram of the culture conditions used to induce chloroplast movement at various temperatures. Gemmalings were pre-cultured under white light (WL) for 1 day and moved to BL25 conditions. **B** Observation of chloroplast positions following incubation under the conditions shown in **A**. Bars represent 50 µm. **C** Quantitative analysis of chloroplast positions shown in **B** based on the P/A ratio. Error bars represent standard deviations (*n* = 5). Different letters indicate statistically significant differences (Tukey’s multiple comparisons test, *P* < 0.05). **D** Diagram of actual temperatures and temperature differences when the temperature decreased from 22 °C to 7 °C

In *M. polymorpha* gemmalings, the accumulation response and the cold-avoidance response are induced under BL25 conditions at 22 °C and 5 °C, respectively (Ogasawara et al. 2013). To further explore the response of chloroplast movement to temperature, we incubated WT gemmalings under BL25 conditions at 22 °C for 1 day followed by 2 °C, 7 °C, 12 °C, 17 °C, or 22 °C for 1 day (Fig. 1A). To detect the accumulation or the cold-avoidance response, we quantified chloroplast position using the P/A ratio method (Kodama et al. 2008). Briefly, this method determines the ratio of chloroplasts (chlorophyll fluorescence) positioned along the periclinal walls (P) to those along the anticlinal walls (A); a higher P/A ratio indicates the accumulation response, and a lower P/A ratio indicates the cold-avoidance response (Fig. S1) (Kodama et al. 2008). The accumulation response was observed at 12 °C, 17 °C, and 22 °C (Fig. 1B and C), whereas the cold-avoidance response was observed at 2 °C and 7 °C (Fig. 1B and C). The degree of the cold-avoidance response was higher at 2 °C than at 7 °C (Fig. 1B and C). These results indicate that in *M. polymorpha* cells under BL25 conditions, when the temperature decreased from 22 °C, the transition between the accumulation response and the cold-avoidance response was triggered between 12 °C and 7 °C. The cold-avoidance response was induced by either sensing actual temperatures (i.e., 7 °C) or by detecting temperature differences (i.e., a 15 °C difference [from 22 °C to 7 °C]) (Fig. 1D).

### Chloroplast movement responds to actual temperatures rather than temperature differences

**Fig. 2:**
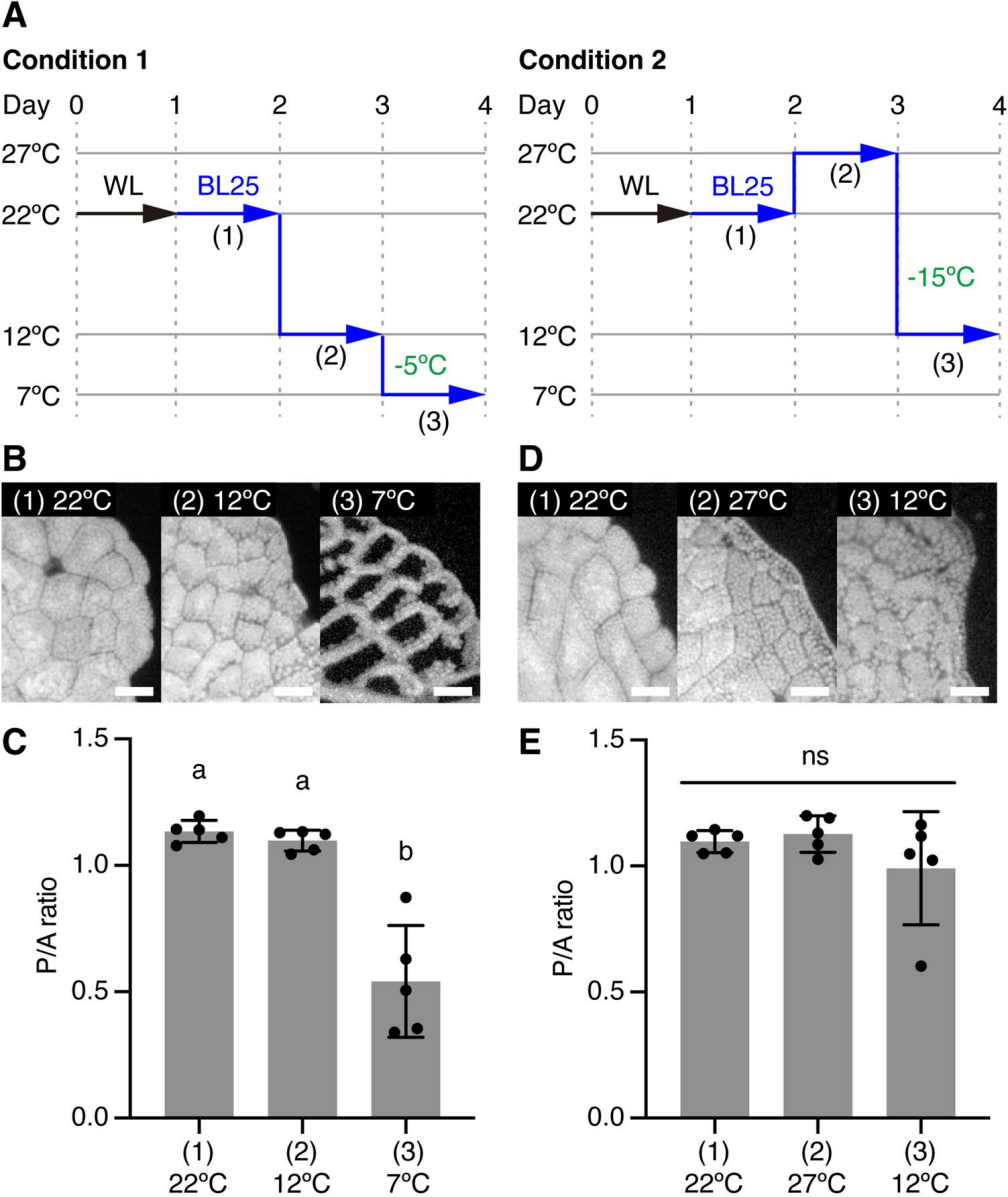
Plant cells sense the actual temperature, not temperature differences. **A** Diagram of Condition 1 and Condition 2 used to induce chloroplast movement. Temperatures shown in green indicate temperature differences. **B** Observation of chloroplast positions following incubation under Condition 1 shown in **A**. Bars represent 50 µm. **C** Quantitative analysis of chloroplast positions shown in **B** based on the P/A ratio. **D** Observation of chloroplast positions following incubation under Condition 2 shown in **A**. Bars represent 50 µm. **E** Quantitative analysis of chloroplast positions shown in **D** based on the P/A ratio. **C** and **E** Error bars represent standard deviations (*n* = 5). Different letters indicate statistically significant differences (Tukey’s multiple comparisons test, *P* < 0.05). ns, not significant

To clarify whether chloroplast movement responds to actual temperatures or temperature differences, we designed a new set of experiments to monitor the cold-avoidance response under BL25 conditions. We incubated WT gemmalings under two temperature conditions: (i) Condition 1, 7 °C with a 5 °C difference (from 12 °C to 7 °C); and (ii) Condition 2, 12 °C with a 15 °C difference (from 27 °C to 12 °C) (Fig. 2A). The cold-avoidance response was induced under Condition 1, but not under Condition 2 (Fig. 2B–E). The cold-avoidance response was also induced under BL25 conditions upon a temperature shift from 27 °C to 7 °C (Fig. S2). These results indicate that incubation at 27 °C does not inhibit the induction of the cold-avoidance response under Condition 2 (Fig. 2B–E). These results indicate that the cold-avoidance response under BL25 conditions was triggered by sensing 7 °C, but not by detecting a 15 °C difference, suggesting that chloroplast movement responds to actual temperatures rather than temperature differences.

### The induction of the cold-avoidance response is promoted by a gradual decrease in temperature to a certain threshold

**Fig. 3:**
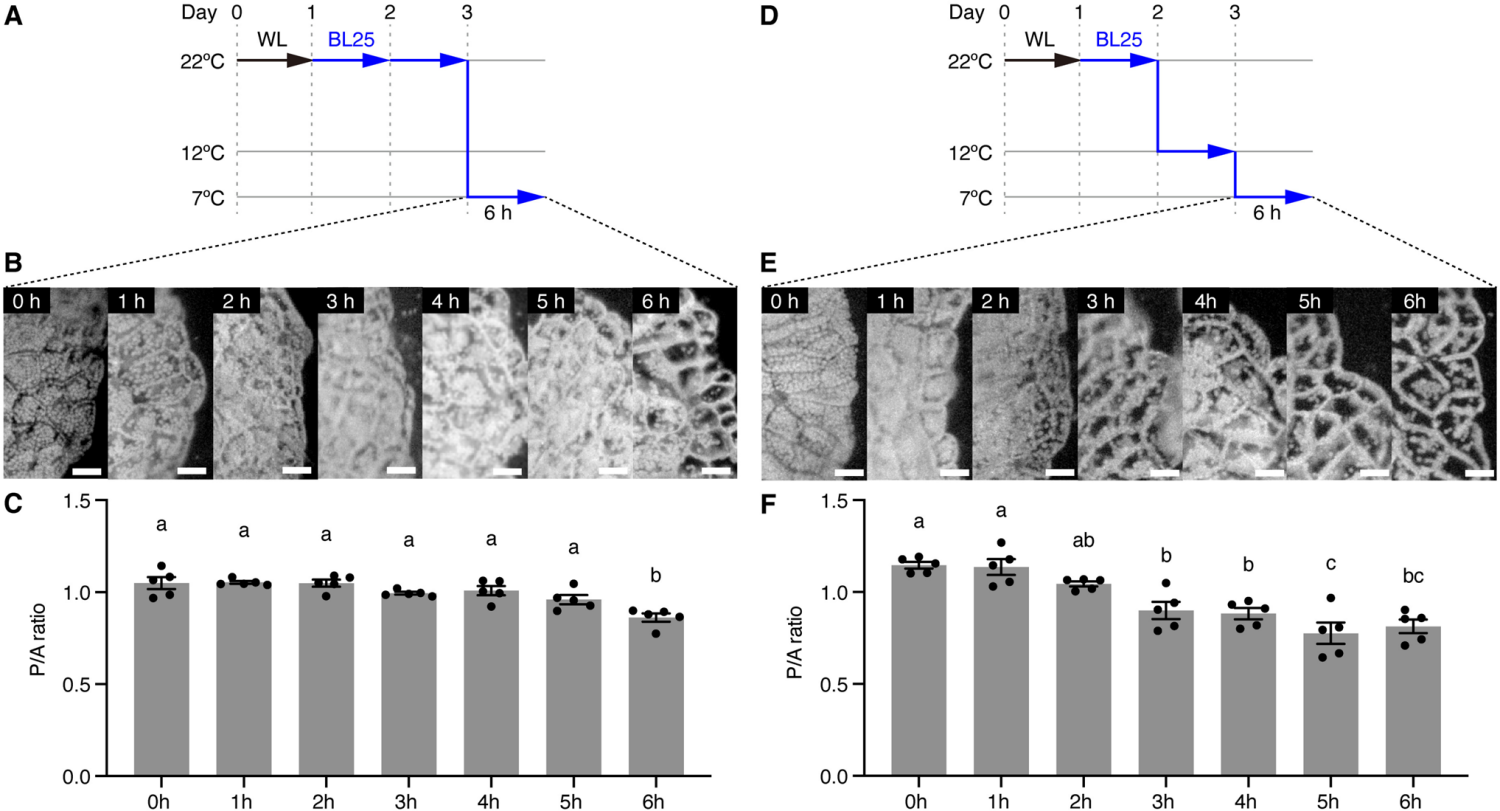
Preincubation at low temperature induces the cold-avoidance response. **A** Diagram of the culture conditions used to induce chloroplast movement with an instantaneous decrease in temperature. **B** Observation of the induction of the cold-avoidance response under BL25 conditions at 7 °C, shown in **A**. Bars represent 50 µm. **C** Quantitative analysis of chloroplast positions shown in **B**, based on the P/A ratio. **D** Diagram of the culture conditions used to induce chloroplast movement with a gradual decrease in temperature. **E** Observation of the induction of the cold-avoidance response under BL25 conditions at 7 °C, shown in **D**. Bars represent 50 µm. **F** Quantitative analysis of chloroplast positions shown in **E**, based on the P/A ratio. **C** and **F** error bars represent standard deviations (*n* = 5). Different letters indicate statistically significant differences (Tukey’s multiple comparisons test, *P* < 0.05)

Next, we investigated whether the cold-avoidance response is induced by an instantaneous or gradual decrease in temperature or both. To create an instantaneous decrease in temperature, we incubated 1-day-old WT gemmalings under BL25 conditions at 22 °C for 2 days to induce the accumulation response, and immediately shifted the temperature from 22 °C to 7 °C to induce the cold-avoidance response (Fig. 3A). Based on microscopy and a comparison of P/A ratios, the cold-avoidance response was detected 6 h after the temperature shift (Fig. 3B and C). To create a gradual decrease in temperature, we incubated 1-day-old WT gemmalings under BL25 conditions at 22 °C for 1 day to induce the accumulation response, followed by incubation at 12 °C for 1 day (to maintain the accumulation response) and a shift to 7 °C to induce the cold-avoidance response (Fig. 3D). During this treatment, the cold-avoidance response was detected 3 h after the temperature shift, and the degree of this response increased thereafter (Fig. 3E and F). These results indicate that a gradual decrease in temperature promotes the cold-avoidance response. We suggest that under BL25 conditions with a temperature range from 22 °C to 12 °C, the cell monitors actual temperatures and modifies its signaling pathways without detectable phenotypic changes in the accumulation response; this "primes" the cell so that when the temperature reaches the threshold to trigger the transition, the cold-avoidance response is induced more quickly.

### Phot senses actual temperatures, not temperature differences, *in planta*

**Fig. 4:**
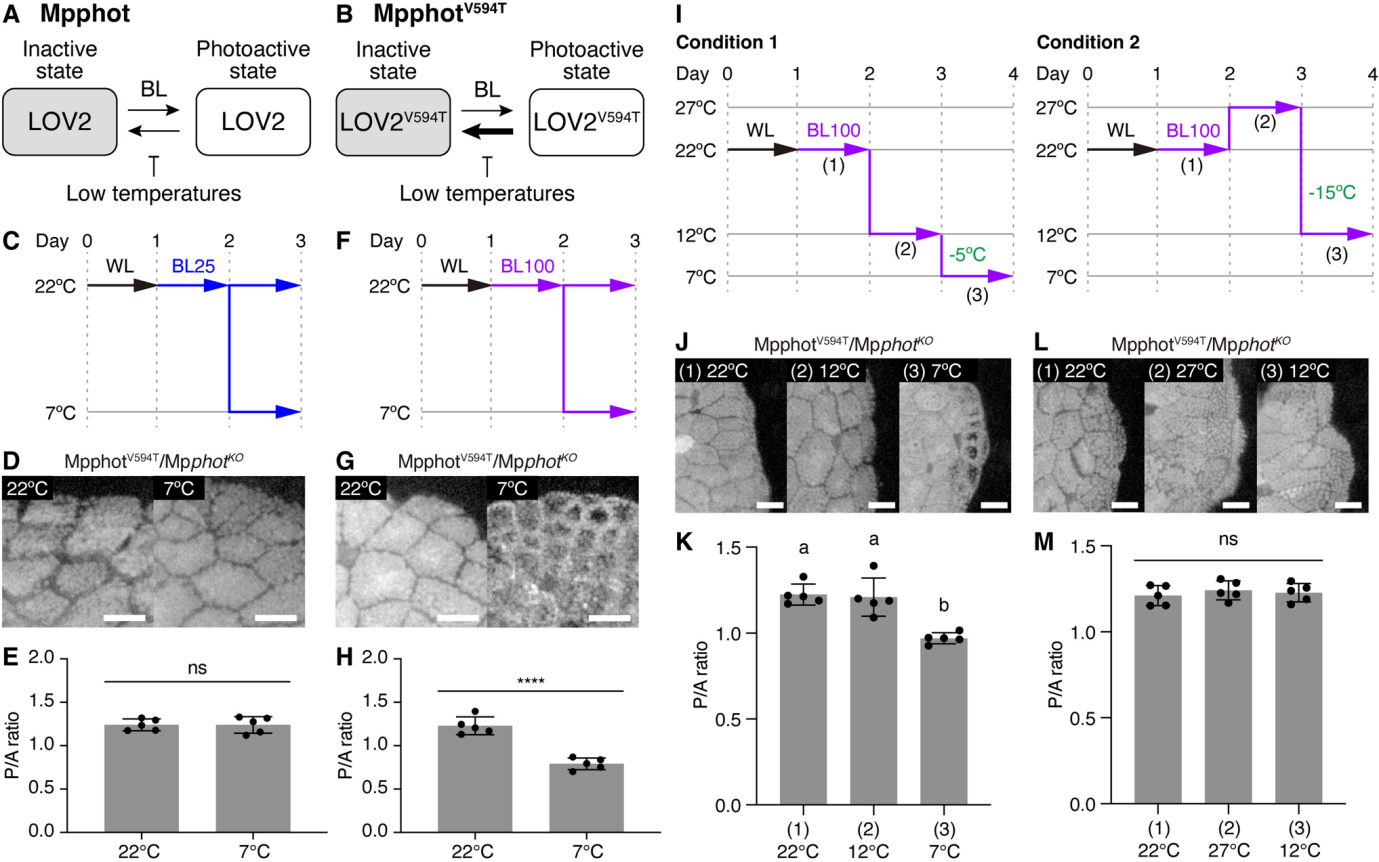
Sensing of the actual temperature to induce the cold-avoidance response depends on thermal reversion of the LOV2 domain in Mpphot. **A** and **B** Diagram of the photocycle of the LOV2 domain in Mpphot (**A**) and Mpphot^V594T^ (**B**). **C** Diagram of the culture conditions used to induce chloroplast movement under BL25 conditions. **D** Observation of chloroplast positions in Mpphot^V594T^/Mp*phot*^*KO*^ under BL25 conditions. **E** Quantitative analysis of chloroplast positions shown in **D** based on the P/A ratio. **F** Diagram of the culture conditions used to induce chloroplast movement under BL100 conditions. **G** Observation of chloroplast positions in Mpphot^V594T^/Mp*phot*^*KO*^ under BL100 conditions. **H** Quantitative analysis of chloroplast positions shown in **G** based on the P/A ratio. **E** and **H** Error bars represent standard deviations (*n* = 5). Asterisks indicate a significant difference in the mean (Student’s *t-*test, ****; *P* < 0.0001). ns, not significant. **I** Diagram of Condition 1 and Condition 2 used to induce chloroplast movement in Mpphot^V594T^/Mp*phot*^*KO*^. Temperatures shown in green indicate temperature differences. **J** Observation of chloroplast positions in Mpphot^V594T^/Mp*phot*^*KO*^ following incubation under Condition 1 shown in **I**. **K** Quantitative analysis of chloroplast positions shown in **J** based on the P/A ratio. **L** Observation of chloroplast positions in Mpphot^V594T^/Mp*phot*^*KO*^ following incubation under Condition 2 shown in **I**. **M** Quantitative analysis of chloroplast positions shown in **L** based on the P/A ratio. **D**, **G**, **J**, and **L** Bars represent 50 µm. **K** and **M** Error bars represent standard deviations (*n* = 5). Different letters indicate statistically significant differences (Tukey’s multiple comparisons test, *P* < 0.05). ns, not significant

Given that temperature-dependent chloroplast movement is triggered by temperature sensing by phot (Fujii et al. 2017), we investigated whether phot senses actual temperatures to induce the cold-avoidance response. Temperature sensing by phot involves the thermal reversion step of the LOV2 domain photocycle: the reversion rate is faster at higher temperatures and slower at lower temperatures (Fujii et al. 2017). Slower rates increase the amount of LOV2 domains in the photoactive state that promote signal transduction including autophosphorylation (Fujii et al. 2017). In a previous study, we modulated the thermosensing ability of Mpphot by substituting threonine for valine at position 594 (V594T) in the LOV2 domain (LOV2^V594T^); the modified LOV2 domain showed a four-fold faster thermal reversion rate than the wild-type LOV2 domain (Fujii et al. 2017) (Fig. 4A and B). Mpphot knockout mutant plants (Mp*phot*^*KO*^) expressing Mpphot^V594T^ (Mpphot^V594T^/Mp*phot*^*KO*^) were less sensitive to low temperatures than WT plants (Fujii et al. 2017).

To confirm that phot senses actual temperatures *in planta*, using thermal reversion of the photocycle of the LOV2 domain, we analyzed the induction of the cold-avoidance response in Mpphot^V594T^/Mp*phot*^*KO*^ cells under various BL and temperature conditions. Similar to our previous observation (Fujii et al. 2017), the accumulation response was induced not only at 22 °C but also at 7 °C in Mpphot^V594T^/Mp*phot*^*KO*^ plants under BL25 conditions (Fig. 4C–E). This result suggests that Mpphot^V594T^/Mp*phot*^*KO*^ plants had insufficient amounts of photoactive LOV2 domains to trigger the cold-avoidance response, leading to a low-temperature-insensitive phenotype (Fig. 4A and B). To increase the amounts of photoactive LOV2 domains, we increased the BL intensity fourfold to 100 µmol m^−2^ s^−1^ (BL100; Fig. 4F). BL100 irradiation rescued the temperature insensitivity phenotype of the Mpphot^V594T^/Mp*phot*^*KO*^ plants: they showed the accumulation response and the cold-avoidance response at 22 °C and 7 °C, respectively (Fig. 4F–H). This result suggests that under BL100 conditions at 7 °C, sufficient amounts of photoactive LOV2 accumulated in Mpphot^V594T^/Mp*phot*^*KO*^ cells to induce the cold-avoidance response.

Similar to the experiment under BL25 shown in Fig. 2, we prepared two temperature conditions under BL100: (i) Condition 1, 7 °C with a 5 °C difference (from 12 °C to 7 °C); and (ii) Condition 2, 12 °C with a 15 °C difference (from 27 °C to 12 °C) (Fig. 4I). The cold-avoidance response was induced in Mpphot^V594T^/Mp*phot*^*KO*^ cells under Condition 1, but not under Condition 2 (Fig. 4I–M). Similar to WT, the cold-avoidance response was also induced under BL100 conditions upon a temperature shift from 27 °C to 7 °C (Fig. S3). These results confirm the notion that phot senses actual temperatures, not temperature differences, and that the cold-avoidance response is induced by a decreased rate of thermal reversion of the photocycle of the LOV2 domain *in planta*.

### The amounts of photoactivated LOV2 domains and autophosphorylated Mpphot gradually increase in response to decreasing temperatures

To investigate the molecular mechanisms associated with phot-mediated sensing of actual temperature, we estimated the amount of photoactivated Mpphot and the autophosphorylation level of Mpphot at various temperatures that induce the accumulation response (12 °C, 17 °C, and 22 °C) or the cold-avoidance response (2 °C and 7 °C) in WT cells under BL25 conditions.

**Fig. 5:**
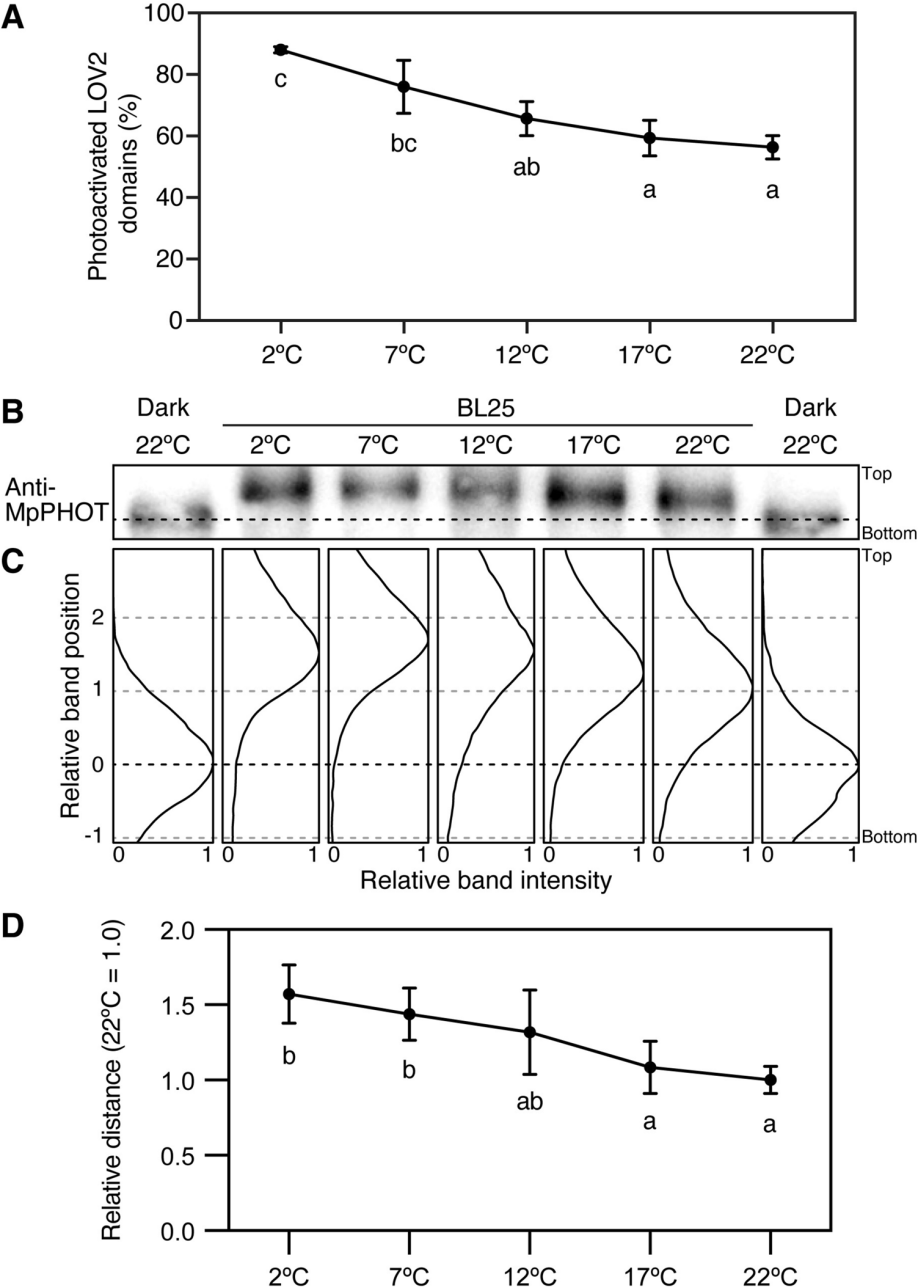
Gradual increases in the amount of the photoactivated LOV2 domain and the autophosphorylation level of Mpphot under decreasing temperatures. **A** The percentage of the photoactivated LOV2 domain under BL conditions in vitro (*n* = 3). Error bars represent standard deviations. Different letters indicate statistically significant differences (Tukey’s multiple comparisons test, *P* < 0.05). **B** Gel mobility shift assay of Mpphot under BL25 conditions. Mpphot was detected by immunoblotting with anti-MpPHOT antibodies. **C** Band intensity histograms of the samples shown in **B**. The band positions of the Dark 22 °C and BL25 22 °C samples were defined as 0 and 1, respectively. **B** and **C** The black dotted lines indicate the band positions of Mpphot in the Dark 22 °C samples. **D** Quantitative analysis of the mobility shift of samples shown in **B** and **C**. The mobility shift assay (**B**) and measurement (**C**) were repeated six times (*n* = 6). The mean distance between Dark 22 °C and BL25 22 °C was defined as 1, and relative distances were calculated. Error bars represent standard deviations. Different letters indicate statistically significant differences (Tukey’s multiple comparisons test, *P* < 0.05)

Because the LOV2 domain is a thermosensing domain that regulates chloroplast movement (Fujii et al. 2017), we calculated the photoactivation rate of Mpphot-LOV2 (recombinant Mpphot-LOV2) in vitro by measuring the fluorescence of inactive LOV2 under BL irradiation (Noguchi et al. 2025a). When we decreased the temperature in 5 °C increments from 22 °C to 2 °C, the photoactivation rate gradually increased (Fig. 5A), which is consistent with the finding that Mpphot perceives actual temperatures through the rate of thermal reversion of the photocycle of the LOV2 domain *in planta* (Fig. 4)*.* To measure the autophosphorylation level of Mpphot under BL25 conditions at 2 °C, 7 °C, 12 °C, 17 °C, or 22 °C *in planta*, we performed a gel electrophoresis mobility shift assay by extracting total proteins from WT gemmalings and separating them using SDS-PAGE (Fujii et al. 2017; Noguchi et al. 2025b). In addition to changes in the photoactivation rate of LOV2 in vitro (Fig. 5A), the autophosphorylation level of Mpphot in vivo gradually increased with decreasing temperature (Fig. 5B–D). These results suggest that in *M. polymorpha* cells, the LOV2 domain of Mpphot senses actual temperatures and that this temperature information is translated into the autophosphorylation level of Mpphot.

### *Trans*-autophosphorylation of Mpphot coincides with induction of the cold-avoidance response

**Fig. 6:**
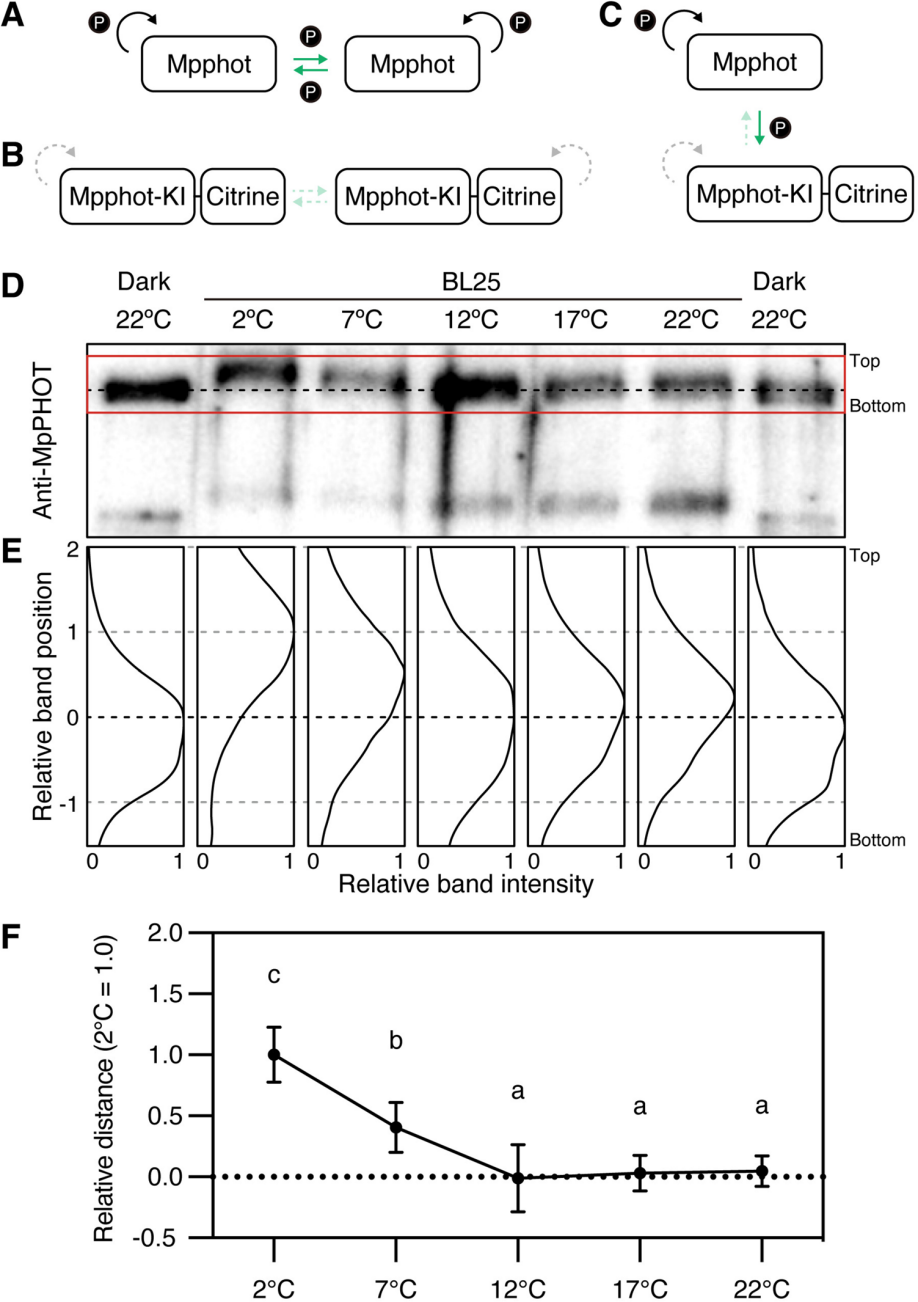
The *trans-*autophosphorylation of Mpphot is crucial for the induction of the cold-avoidance response. **A**–**C** Diagrams of *cis-* and *trans-*autophosphorylation of endogenous Mpphot (**A**), Mpphot-KI-Cit (**B**), and endogenous Mpphot and Mpphot-KI-Cit (**C**). The black and green arrows indicate *cis-* and *trans-*autophosphorylation, respectively. The dashed arrows indicate no phosphorylation. **D** Gel shift assay of Mpphot-KI-Cit in Mpphot-KI-Cit/WT #1 under BL25 conditions to analyze the *trans-*autophosphorylation of Mpphot. Mpphot-KI-Cit was detected by immunoblotting with anti-MpPHOT antibodies. **E** Band intensity histograms of the samples shown in the red rectangle in **D**. The band positions of the Dark 22 °C and BL25 2 °C samples were defined as 0 and 1, respectively. **F** Quantitative analysis of the mobility shift of the samples shown in **D** and **E**. The mobility shift assay (**D**) and measurement (**E**) were repeated seven times (*n* = 7). The mean distance between Dark 22 °C and BL25 2 °C was defined as 1, and relative distances were calculated. Error bars represent standard deviations. Different letters indicate statistically significant differences (Tukey’s multiple comparisons test, *P* < 0.05). **D**–**F** The black dotted lines indicate the band positions of Mpphot-KI-Cit in the Dark 22 °C samples

Although the amount of photoactivated LOV2 domain and the autophosphorylation level of Mpphot gradually changed in a temperature-dependent manner (Fig. 5), it was unclear how these changes trigger the transition between the accumulation response (at 12 °C, 17 °C, and 22 °C) and the cold-avoidance response (at 2 °C and 7 °C) under BL25 conditions (Fig. 1A–C). We recently determined that Mpphot has two modes of autophosphorylation: *cis-*autophosphorylation, which occurs within a single molecule; and *trans-*autophosphorylation mediated by intermolecular interactions (Noguchi et al. 2025b) (Fig. 6A). In our previous study, to distinguish between *cis-* and *trans-*autophosphorylation of Mpphot, we transformed WT cells with a Citrine-fused kinase-inactive Mpphot mutant (Mpphot-KI-Cit, Fig. 6B) to generate Mpphot-KI-Cit/WT lines (Noguchi et al. 2025b). In these Mpphot-KI-Cit/WT lines, the level of phosphorylated Mpphot-KI-Cit can be used as an indicator of the *trans*-autophosphorylation activity of the endogenous Mpphot, as it can only be phosphorylated by endogenous Mpphot in *trans* (Noguchi et al. 2025b) (Fig. 6C). We previously showed that under BL25 conditions, *trans*-autophosphorylation occurs at 5 °C, but not at 22 °C, and that the *trans-*autophosphorylation of Mpphot is required to induce the cold-avoidance response (Noguchi et al. 2025b).

To examine the transition between the two modes of autophosphorylation under BL25 conditions at 2 °C, 7 °C, 12 °C, 17 °C, or 22 °C, we quantified the autophosphorylation level of Mpphot-KI-Cit in Mpphot-KI-Cit/WT #1. At 2 °C and 7 °C, which induce the cold-avoidance response, *trans*-autophosphorylation of Mpphot was detected (Fig. 6D–F). At 12 °C, 17 °C, or 22 °C, which induce the accumulation response, no *trans*-autophosphorylation of Mpphot was detected (Fig. 6D–F). The increase in the *trans*-autophosphorylation level of Mpphot (Fig. 6D–F) coincides with the induction of the cold-avoidance response (Fig. 1A–C), suggesting that the transition from the accumulation response to the cold-avoidance response is due to the change in the mode of autophosphorylation from *cis* to *trans*.

## Discussion

In this study, we demonstrated that Mpphot senses actual temperatures, rather than relative temperature changes, to induce the cold-avoidance response in *M. polymorpha* cells (Fig. 2). The thermal reversion of the photocycle of the LOV2 domain in Mpphot plays a crucial role in actual temperature sensing (Fig. 4). The amount of the photoactivated LOV2 domain and the autophosphorylation level of Mpphot gradually increased in response to decreasing temperatures (Fig. 5). Furthermore, *trans*-autophosphorylation of Mpphot coincides with the induction of the cold-avoidance response (Fig. 1 and Fig. 6).

Chloroplast movement is a bidirectional reaction in which chloroplasts move toward or away from the light-irradiated region of the cell depending on BL intensity and temperature (Kodama et al. 2008; Wada 2013). At standard growth temperatures, chloroplasts move away from areas of the cell irradiated with strong BL, thereby reducing light intensity-dependent photoinhibition, a process known as the high light (HL)-avoidance response (Kasahara et al. 2002). The HL-avoidance response is induced when the intensity of the BL irradiation exceeds a certain threshold. We previously proposed a model in which the threshold BL intensity for switching between the accumulation and HL-avoidance responses shifts depending on ambient temperatures (Fujii et al. 2020). In the present study, we evaluated chloroplast positions using the P/A ratio method, which provides high-throughput and robust quantification of chloroplast positioning (Kodama et al. 2008, 2010; Fujii et al. 2017; Tanaka et al. 2017). Using this approach, we showed that the threshold temperature for the transition from the accumulation response to the cold-avoidance response could be shifted by changes in BL intensity (Fig. 4). Therefore, for the transition between the accumulation and avoidance responses (under cold and HL conditions), the BL intensity threshold depends on the temperature—not limited to low temperature—, whereas the temperature threshold depends on the BL intensity.

We previously identified 23 serine and threonine phosphorylation sites in Mpphot (Noguchi et al. 2025b), which appeared to be randomly phosphorylated under three different environmental conditions (dark at 22 °C, BL25 at 22 °C, and BL25 at 5 °C) (Noguchi et al. 2025b). Importantly, Mpphot has two modes of autophosphorylation: *cis-* and *trans*-autophosphorylation (Noguchi et al. 2025b). The *cis*-autophosphorylation of Mpphot helps to induce the accumulation response (Noguchi et al. 2025b). In addition to *cis*-autophosphorylation, *trans*-autophosphorylation is required to increase Mpphot autophosphorylation levels and to induce the cold-avoidance response (Noguchi et al. 2025b). Since enzyme activity generally decreases at low temperatures, the intrinsic enzyme activity of the kinase domain in Mpphot may also decrease at low temperatures. However, under BL conditions, the autophosphorylation level of phot increases at lower temperatures in vivo and in vitro (Okajima et al. 2012; Fujii et al. 2017). The inactive LOV2 domain interacts with the kinase domain to inhibit enzymatic activity, whereas the photoactive LOV2 domain dissociates from the kinase domain, thereby releasing the inhibition. Under low-temperature conditions, the photoactivated state of LOV2 is maintained, leading to increased autophosphorylation (Fujii et al. 2017). Here we showed that the autophosphorylation level of Mpphot under BL25 conditions increased gradually in response to decreasing temperature (Fig. 5B–D). Because *trans*-autophosphorylation of Mpphot was not induced at $\ge$ 12 °C (Fig. 6), it appears that the gradual increase in the autophosphorylation level of Mpphot under BL25 conditions at 22 °C, 17 °C, and 12 °C is induced only by *cis-*autophosphorylation (Fig. 5B–D). On the other hand*, trans*-autophosphorylation of Mpphot was observed at $\le$ 7 °C. At these low temperatures, *trans*-autophosphorylation of Mpphot may serve to compensate for reduced kinase activity through intermolecular interactions that are stabilized at low temperatures.

To date, the mechanism by which autophosphorylation transitions from the *cis* to the *trans* mode remains to be determined. Given that phosphorylation of the 23 sites in Mpphot occurs randomly (Noguchi et al. 2025b), it is unlikely that phosphorylation of a specific residue triggers the change in activity. Because *trans*-autophosphorylation follows *cis*-autophosphorylation (Fig. 5B–D and Fig. 6) (Noguchi et al. 2025b), there may be a threshold for the total number of phosphorylated residues on phot, beyond which the kinase domain transitions from the *cis* to the *trans* mode of autophosphorylation. An increasing number of phosphorylated residues through *cis*-autophosphorylation appears to induce structural changes in Mpphot. When this number reaches the threshold, the structurally altered Mpphot may interact with other Mpphot molecules, leading to the switch from *cis*- to *trans*-autophosphorylation. Conversely, if the number of phosphorylated residues falls below the threshold through dephosphorylation, the intermolecular interaction would be abolished. We hypothesize that such autophosphorylation-mediated, reversible structural regulation of Mpphot functions as a switch between the accumulation and cold-avoidance responses.

Although the signal transduction pathways for the cold-avoidance response are still unknown, *trans*-autophosphorylation of Mpphot is necessary for the cold-avoidance response, but not for the accumulation response (Noguchi et al. 2025b), suggesting that the signal transduction pathways for the accumulation and cold-avoidance responses differ. Based on the current data (Fig. 6), *trans-*autophosphorylation of Mpphot is likely involved in determining the threshold temperature for switching between the accumulation and cold-avoidance responses (Fig. 6). As *trans*-autophosphorylation occurs through intermolecular interactions between Mpphot proteins, the transition from monomeric to oligomeric forms of Mpphot may shift signaling from the accumulation response to the cold-avoidance response via interactions with other proteins: its dissociation from proteins involved in the accumulation response and/or its interaction with proteins required for inducing the cold-avoidance response. Of note, the plasma membrane-localized Mpphot functions as a thermosensor molecule (Hirano et al. 2022). Further analysis of the relationship between the autophosphorylation modes of Mpphot and its interacting proteins at the plasma membrane could help uncover the switching mechanism between the accumulation response and the avoidance response.

In the present study, we showed that a gradual decrease in temperature promotes the cold-avoidance response in *M. polymorpha* when temperatures reach the threshold that triggers this response (Fig. 3). Since temperature conditions change gradually in nature, the cold-avoidance response might represent an acclimation to natural temperature variations. When temperature gradually decreases, plant cells might begin preparing the cold-avoidance response before reaching the threshold temperature for switching between the accumulation and cold-avoidance responses. Temperature sensing by phot was shown to mediate chloroplast movement in *M. polymorpha* and Arabidopsis and to mediate phototropism and stomatal opening in Arabidopsis (Fujii et al. 2017; Noguchi et al. 2025a). Arabidopsis contains two copies of phot (Atphot1 and Atphot2): Atphot2 functions as a thermosensor to initiate BL responses (Fujii et al. 2017; Noguchi et al. 2025a). Since phototropism and stomatal opening are not bidirectional responses like chloroplast movement, they are not considered to be threshold temperature-dependent reactions. However, given that these BL responses contribute to optimizing photosynthetic activity and are primed by the low-temperature treatments (Christie 2007; Noguchi et al. 2025a), temperature sensing by phot for BL responses may share common characteristics. Similar to the response of chloroplast movement in *M. polymorpha* to a gradual decrease in temperature (Fig. 3), chloroplast movement, phototropism, and stomatal opening are also thought to respond to a gradual change in temperature in Arabidopsis.

Protein phosphorylation levels are determined through a balance of phosphorylation and dephosphorylation by kinases and phosphatases, respectively. In the case of phot, phosphorylation is mediated by its kinase activity (i.e., autophosphorylation). In this study, we demonstrated that the autophosphorylation level of Mpphot under BL25 conditions gradually increased in response to decreasing temperature (Fig. 5 B–D). A previous in vitro study also showed that the autophosphorylation levels of both Atphot1 and Atphot2 gradually increased upon a decrease in temperature (Okajima et al. 2012). For dephosphorylation by phosphatases in Arabidopsis, the A1 subunit of serine/threonine protein phosphatase 2 A directly interacts with Atphot2 and is involved in the regulation of Atphot2-mediated BL responses (Tseng and Briggs 2010). Although phosphatases involved in chloroplast movement in *M. polymorpha* are still unknown, the autophosphorylation level of Mpphot in vitro rapidly decreased in the dark (Komatsu et al. 2014), suggesting the existence of phosphatases that act on Mpphot. Given that enzyme activity generally declines at low temperatures, the increased autophosphorylation levels of phot at low temperatures, via LOV2-mediated unlocking of the kinase domain, may be promoted by reduced phosphatase activity at low temperatures.

In the present study, we determined that Mpphot senses actual temperatures, rather than temperature differences (Fig. 4 and Fig. 5). Similar to the finding that Mpphot undergoes thermosensing via thermal reversion of the LOV2 domain for chloroplast movement (Fujii et al. 2017), the thermosensing of Atphot2 for stomatal opening and phototropism also depends on thermal reversion of its LOV2 domain (Noguchi et al. 2025a). Considering that both Mpphot and Atphot2 sense temperatures using the same mechanism, perhaps Atphot2 senses actual temperatures rather than relative temperature differences in order to optimize BL responses in Arabidopsis. We previously hypothesized that many photoreceptors might function as thermosensors based on the thermal reversion of their photocycles (Fujii et al. 2017; Noguchi and Kodama 2022). For example, the red/far-red light receptor phytochrome B (phyB) also functions as a thermosensor via thermal reversion in Arabidopsis (Jung et al. 2016; Legris et al. 2016). Phot and phyB function as thermosensors for low and high temperature ranges, respectively (Jung et al. 2016; Legris et al. 2016; Fujii et al. 2017; Noguchi and Kodama 2022). Because phyB is a thermal reversion-based thermosensor as well, it may also sense actual temperatures rather than relative temperature differences. However, we cannot exclude the possibility that phot and phyB sense the latter. Recently, it was reported that the N-terminal extension of phyB functions as a thermosensing domain through the formation of liquid–liquid phase separation (LLPS) (Chen et al. 2022). Furthermore, the interactions between phyB and PHYTOCHROME INTERACTING FACTOR 6 (PIF6) prolong the lifetime of photoactivated phyB and regulate phyB LLPS formation, suggesting that the interaction with PIF6 regulates phyB thermosensing (Wang et al. 2024; Jia et al. 2025). These findings indicate that photoreceptor-mediated thermosensing is regulated by complex mechanisms involving not only the thermal reversion but also interactions with other proteins. It remains unclear whether the interaction-based mechanism senses actual temperatures or relative temperature differences. Further studies to address these questions are needed to fully understand temperature-sensing mechanisms in plants.

## Supplementary Information

Below is the link to the electronic supplementary material.

Supplementary file1 (DOCX 8798 KB)

## Data Availability

The authors declare that all data supporting the finding of this study are available within the manuscript or are available from the corresponding author upon request.

## Abbreviations

BL: Blue light
Cit: Citrine
KI: Kinase inactive
LOV: Light-oxygen-voltage
phot: Phototropin
wBL: Weak blue light
