# Supplementary material for: Phototropin monitors actual temperature, not temperature difference, to regulate temperature-dependent chloroplast movement via cis–trans autophosphorylation mode switching in Marchantia polymorpha
Source: Planta. 2026 Jan 17;263(2):55. doi: 10.1007/s00425-026-04923-1 (PMC12812097; doi:10.1007/s00425-026-04923-1)
Supplement: Supplementary file 1 — Supplementary file1 (DOCX 8798 KB) [file 425_2026_4923_MOESM1_ESM.docx]

**
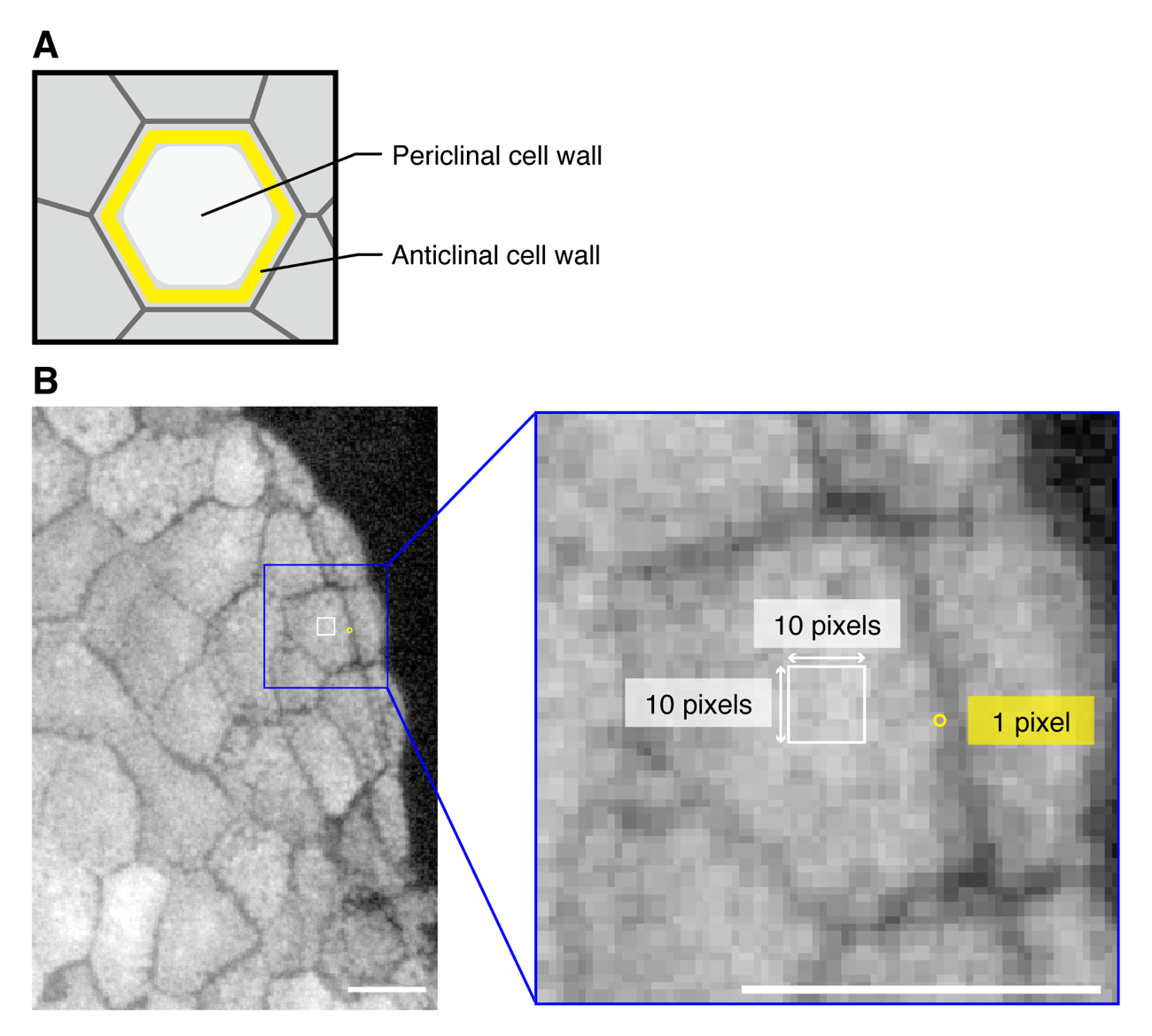
Fig. S1** Quantification of chloroplast positioning using the P/A ratio method. **A** Diagrams of periclinal and anticlinal cell wall areas. **B** Representative images used to measure fluorescence intensity at the periclinal (white square) and anticlinal (yellow circle) cell wall areas. To quantify the intensity at the periclinal cell wall, we calculated the average fluorescence intensity within a 10 × 10-pixel region of interest. The chlorophyll fluorescence intensity at the anticlinal cell wall was quantified by measuring the fluorescence intensity of a single pixel. We observed 30 cells in each gemmaling to calculate the average P/A ratio and repeated this analysis for five gemmalings (*n* = 5). Bars represent 50 µm

**Fig. S2** The cold-avoidance response is induced in WT gemmalings under BL25 conditions upon a temperature shift from 27°C to 7°C. **A** Diagram of the culture condition used to induce chloroplast movement in WT cells. **B** Observation of chloroplast positions in WT cells following incubation under the condition shown in **A**. Bars represent 50 µm. **C** Quantitative analysis of chloroplast positions shown in **B** based on the P/A ratio. Error bars represent standard deviations (*n* = 5). Different letters indicate statistically significant differences (Tukey’s multiple comparisons test, *P* < 0.05)**
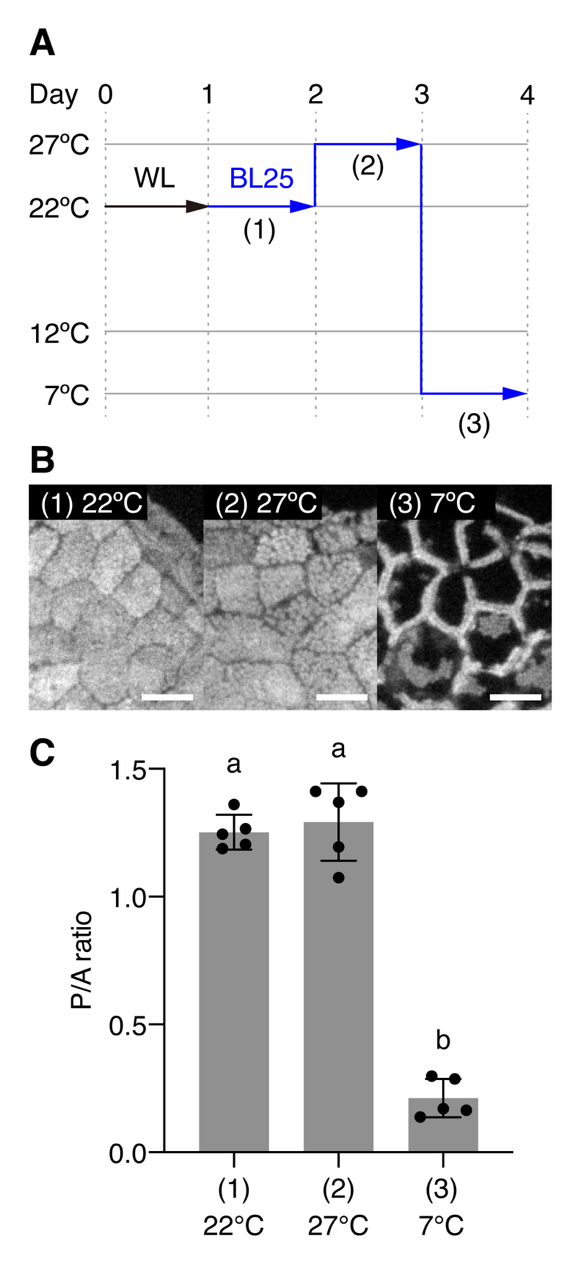
**

**
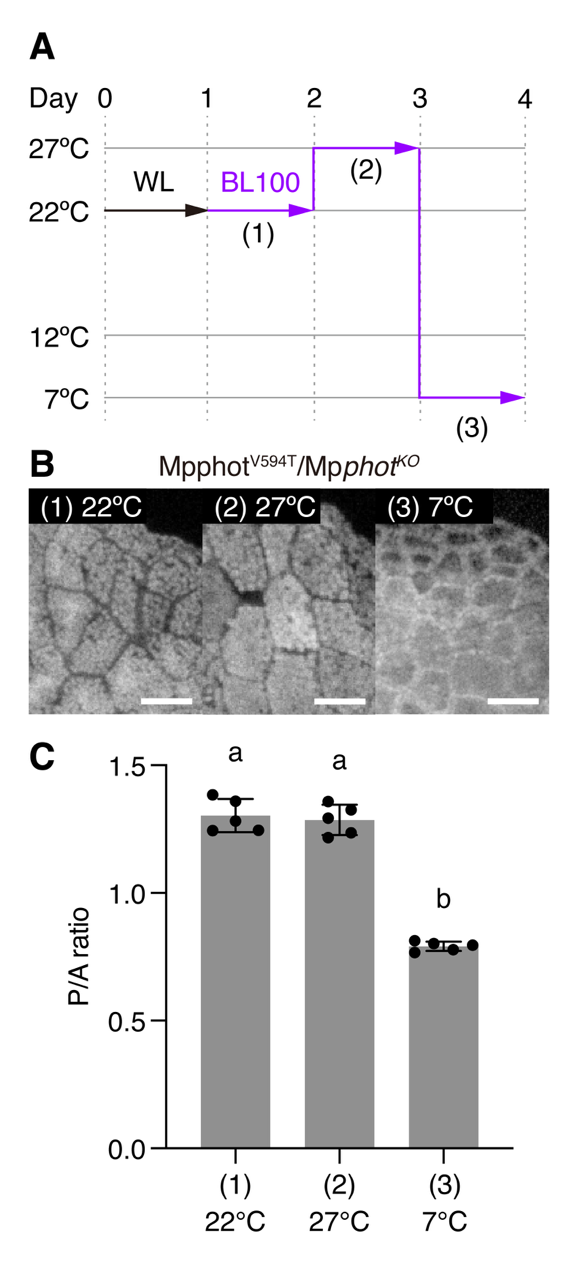
Fig. S3** The cold-avoidance response is induced in the Mpphot^V594T^/Mp*phot^KO^* cells under BL100 conditions upon a temperature shift from 27°C to 7°C. **A** Diagram of the culture condition used to induce chloroplast movement in Mpphot^V594T^/Mp*phot^KO^*. **B** Observation of chloroplast positions in Mpphot^V594T^/Mp*phot^KO^* cells following incubation under the condition shown in **A**. Bars represent 50 µm. **C** Quantitative analysis of chloroplast positions shown in **B** based on the P/A ratio. Error bars represent standard deviations (*n* = 5). Different letters indicate statistically significant differences (Tukey’s multiple comparisons test, *P* < 0.05)
